# An unusual case of recurrent Guillain-Barré syndrome with normal cerebrospinal fluid protein levels: a case report

**DOI:** 10.1186/s12883-016-0687-z

**Published:** 2016-09-05

**Authors:** Sonali Sihindi Chapa Gunatilake, Rohitha Gamlath, Harith Wimalaratna

**Affiliations:** Teaching Hospital, Kandy, Sri Lanka

**Keywords:** Guillain-Barré syndrome, Albuminocytologic dissociation, Acute inflammatory demyelinating polyneuropathy, Cerebrospinal fluid, Case report

## Abstract

**Background:**

Guillain-Barré syndrome is an acquired polyradiculo-neuropathy, often preceded by an antecedent event. It is a monophasic disease but a recurrence rate of 1–6 % is documented in a subset group of patients. Patients with Guillain-Barré syndrome show cerebrospinal fluid albuminocytologic dissociation. Normal cerebrospinal fluid protein levels during both initial and recurrent episodes of Guillain-Barré syndrome is a rare occurrence and has not been described earlier in the literature.

**Case presentation:**

Twenty-five-year-old Sri Lankan female with past history of complete recovery following an acute inflammatory demyelinating polyneuropathy (AIDP) variant of Guillain-Barré syndrome 12 years back presented with acute, ascending symmetrical flaccid quadriparasis extending to bulbar muscles, bilateral VII cranial nerves and respiratory compromise needing mechanical ventilation. Nerve conduction study revealed AIDP variant of Guillain-Barré syndrome. Cerebrospinal fluid analysis done after 2 weeks were normal during both episodes without albuminocytologic dissociation. She was treated with intravenous immunoglobulin resulting in a remarkable recovery. Both episodes had a complete clinical recovery in three and four months’ time respectively, rather a faster recovery than usually expected.

**Conclusion:**

Recurrence of Guillain-Barré syndrome can occur in a subset of patients with Guillain-Barré syndrome even after many years of asymptomatic period. Normal cerebrospinal fluid profile does not exclude Guillain-Barré syndrome and may occur in subsequent recurrences of Guillain-Barré syndrome arising the need for further studies to identify the pathophysiology and the possibility of a different subtype of Guillain-Barré syndrome.

## Background

Guillain-Barré syndrome (GBS) is an acquired heterogeneous group of disorders due to an immune-mediated inflammation and demyelination of the peripheral nervous system, following an antecedent illness in two thirds of the patients, commonly an infection [1–3]. It is a medical emergency which usually presents with acute onset, rapidly progressive symmetrical ascending flaccid paralysis of the limbs with accompanying absent or diminished deep tendon reflexes. It is often associated with sensory symptoms, cranial nerve involvement, less commonly autonomic dysfunction and respiratory compromise.

GBS is a monophasic illness. Although rare, recurrence has been described following an asymptomatic period of few months to years (4 months – 10 years) in 1–6 % of patients [3–6]. Recurrent GBS (RGBS) is characterized by 2 or more attacks of acute inflammatory demyelinating neuropathy with an onset to peak time of 4 weeks or less, and having complete or near complete recovery [3, 5]. It is suggested by Kuitwaard et al. that there is a subset of patients with GBS who are susceptible for recurrence, characterized by younger age, milder course of disease and having Miller-Fisher variant of GBS [3]. Literature revealed that the patients with recurrence had similar but more severe symptoms and signs in subsequent episodes while having similar or different antecedent event [3, 6]. It is important to distinguish recurrent GBS from GBS with treatment related fluctuations (GBS-TRF) and chronic inflammatory demyelinating polyradiculo-neuropathy (CIDP) as the treatment regimens are different. Cerebrospinal fluid (CSF) shows albuminocytologic dissociation in 82–90 % of the patients with GBS after 10–14 days from onset of the illness [7]. Electrophysiological studies and CSF analysis are taken to aid clinical diagnosis of GBS but normal CSF profile can be found in 10 % of GBS patients throughout the disease [8]. Therefore normal values cannot rule out GBS. Grand’maison et al. reported 12 cases of recurrent GBS (total of 32 episodes), where it was observed that all patients who were in the symptomatic phase of GBS and after 1 week of onset of disease showed CSF albuminocytologic dissociation. They observed normal CSF protein levels which was tested at the onset of disease (within 1 week) in two patients but had not identified a variant of RGBS with normal CSF protein levels during the symptomatic episodes, tested atleast 1 week after the onset of symptoms.

We present a rare case of RGBS presenting after 12 years, adding to the limited number of cases with a long asymptomatic interval. Such reported cases from South-Asia are rare. Apart from the young age at initial episode, she did not have other risk factors for recurrence and had a rare findings of normal CSF protein concentrations on both presentations. Extensive literature survey including published case reports and case series did not reveal a similar case report of RGBS or occurrence with normal CSF protein concentrations during both initial and recurrent presentations of GBS.

## Case presentation

A 25-year-old Sri Lankan female presented with weakness of all four limbs in January 2014. She had a similar illness 12 years back.

### First episode

In 2002, at the age of 13 years, patient had noticed tingling sensation of distal upper and lower limbs followed by weakness of lower limbs, involving both distal and proximal muscle groups. Weakness was progressive and ascending, involving both upper limbs and neck muscles by day 6 of the illness. There was no dysphagia, dysphonia, respiratory difficulty or bladder/bowel involvement. There was no significant medical history suggestive of preceding infection, toxin ingestion or similar disease in the past. On examination, there was flaccid quadriparesis (muscle power grade - 3/5) with areflexia in all 4 limbs. There were no cranial nerve palsies or features suggestive of autonomic involvement. Sensory system examination was normal. Full blood count, blood picture, serum electrolytes, blood urea and liver enzymes profile were normal. Her erythrocyte sedimentation rate was 13mm during 1^st^ hour. Nerve conduction studies performed on day 4 of the illness showed focal segmental demyelinating type sensory and motor neuropathy with conduction blocks (Table 1), suggestive of acute inflammatory demyelinating polyneuropathy (AIDP). EMG studies were not performed during this episode. CSF analysis on day 14 of the illness revealed normal results with proteins – 30 g/dl (normal 15–40 g/dl), white cells 2 with 100 % lymphocytes and normal CSF glucose levels compared to plasma values. She was treated with intravenous immunoglobulin for 5 consecutive days in addition to physiotherapy and discharged on day 18. She made a complete recovery in 3 months based on normal tone, power and deep tendon reflexes on neurological examination. Follow-up NCS was not performed after clinical recovery.

### Second episode

In 2014, patient readmitted with numbness and progressive ascending weakness of all four limbs for 3 days duration and had developed poor cough response, dysphagia and difficulty in breathing during the following 2 days. This episode was preceded by an upper respiratory tract infection two weeks back. There was no similar illness noted in any of her family members. On examination, flaccid quadriparesis with generalized areflexia was noted with a muscle power of 1/5 in lower limbs, 2/5 in upper limbs and 2/5 in neck muscles. Weakness progressed to involve bilateral seventh cranial nerves and bulbar muscle without ophthalmoplegia. Rest of the neurological examination including sensory system and other organ system examination were normal except for a resting tachycardia of 130 beats/min without any significant blood pressure fluctuations. Respiratory rate was 30 cycles/min with oxygen saturation of 92 % on air. Nerve conduction studies done on day 10 of the illness revealed focal segmental demyelinating type sensory and motor neuropathy with prolonged distal motor latency, delayed F-wave and conduction blocks, concluding as AIDP variant of GBS (Table 1). Electromyogram done on day 10 of the illness did not show any evidence of denervation but noted fibrillation potentials of positive sharp waves. CSF analysis on day 10 and a repeat study on day 24 showed normal results without cyto-protein dissociation or pleocytosis (Table 2) Full blood count, blood picture, serum electrolytes, erythrocyte sedimentation rate, blood urea and liver enzymes profile were normal. Serology for *Mycoplasma, Campylobacter jejuni*, cytomegalovirus, Epstein-Barr virus, hepatitis B & C and retroviral studies were negative. Autoimmune panel including anti-nuclear factor was normal. Due to the rapid progressive nature of weakness and respiratory distress, patient was mechanically ventilated for 12 days. Diagnosis of GBS was made and intravenous immunoglobulin 0.4 g/kg/day was administered for 5 days. In addition, she received physiotherapy to support her recover in motor function of limbs and speech therapy following extubation. She made a good clinical recovery assessed subjectively as well as objectively and was discharged home on day 28 with a muscle power of 4/5 and deep tendon reflexes of +1.

**Table 1:** Nerve conduction study during the initial presentation in 2002 (1^st^ episode) and recurrence (2^nd^ episode) of Guillain-Barre syndrome in 2014

Nerve	Episode	Distal latency (ms)	Conduction velocity (m/s)	Amplitude (mV)	F wave (m/s)	Conduction block
Motor						
Median (Right/left)	1^st^	6.7/6.2	40/45	7.5/11	33/35	Yes
	2^nd^	9.1/8.9	38/40	5.6/ 6	38/40	Yes
	Recovery (4 months after 2^nd^ Episode)	4.4/4.5	51	6.0	29	No
	Normal Referrence Value	<4.4	>49	>4.2	<31	
Ulnar (Right/left)	1^st^	4.6/5.0	42/44	6.0/ 6.2	27/32	Yes
	2^nd^	8.9/9.4	37/41	8.2/ 1.5	36/29	Yes
	Normal Referrence Value	<3.5	>49	>5.6	<31	
Peroneal @ EDB (Right/left)	1^st^	11.5/13	38/45	2.5/ 2.4	43/49	NP
	2^nd^	Absent	Absent	1.6/2.0	Absent	Yes
	Recovery (4 months after 2^nd^ Episode)	5.5	50	2.5	33	No
	Normal Referrence Value	<5.7	>50	>2.2		
Tibial (Right/left)	1^st^	18.8/18.9		3.0/3.2	52/49	NP
	2^nd^	NP	NP	NP	NP	NP
	Normal Referrence Value			>2.8		
Sensory						
Radial (Right/left)	1^st^	2.8/ 3.1	38/37	24/23	-	-
	2^nd^	2.9/ 3.0	28/33	18/20		
	Normal Referrence Value	<2.6	>43	>20		
Sural (Right/left)	1^st^	2.5/3.0	41/40	34/28		
	2^nd^	2.6/2.5	46/48	25/27	-	-
	Normal Referrence Value	<2.6	>52	>24		

*NP* not performed

*EDB* Extensor digitorum brevis

**Table 2:** Laboratory findings of Cerebrospinal fluid during the initial presentation in 2002 and recurrence of Guillain-Barré syndrome in 2014

Cerebrospinal fluid profile	2002 episode: 1 – Day 14	2014 recurrence – Day 10	2014 recurrence – Day 24
CSF Glucose (mg/dL)	86	78	80
Proteins (g/dL) (normal: 15 – 40 g/dL)	30	20	26
White blood cells/HPF (normal: 0 – 5 cells/HPF)	2	4	2
Neutrophils %	-	-	-
Lymphocytes %	100	100	100
Red blood cells/HPF	5	-	10
Random blood glucose tested at the time of lumba puncture (mg/dL)	102	110	94

CSF study was performed twice during the episode of recurrence (2014) to clarify the persistence of normal CSF findings during the course of the illness

On follow-up, patient had normal neurological examination findings and subsequent nerve conduction study after 4 months revealed normal results (Table 1).

## Conclusions

GBS is an acute, immune mediated inflammatory polyradiculo-neuropathy involving the peripheral nervous system. Onset is preceded by an antecedent event in two thirds of the patients, usually an upper respiratory tract infection or a diarrheal illness [1–3], where the causative agent is assumed to trigger an immune response against the gangliosides and glycolipids distributed along the myelin sheaths and peripheral nervous system. This results in marked inflammation of the peripheral nerves, resulting in demyelination and defective impulse propagation. It is a heterogeneous group of disorders which involves motor, sensory and autonomic nervous systems to varying degrees depending on the sub type; (1) Acute inflammatory demyelinating polyneuropathy, (2) Acute motor axonal neuropathy, (3) Acute motor sensory axonal neuropathy, (4) Miller Fisher syndrome, (5) Acute pan-autonomic neuropathy and (6) Pure sensory GBS.

GBS is a monophasic illness, with an annual incidence rate of 1.2–3 per 100 000 population [9]. Yet, recurrence of GBS is observed in 1–6 % of patients, where it is defined as 2 or more attacks of acute inflammatory demyelinating neuropathy with an onset to peak time of 4 weeks or less having complete or near complete recovery [3–6]. The time lag between two episodes of GBS was 4 months to 10 years in a study done by Das et al. and a mean of 7 years with a range from 2 months to 37 years was described by Kuitwaard et al. [3, 5]. Patients tend to get similar clinical presentations and shorter intervals in between subsequent episodes of GBS [3]. Results of the study by Kuitwaard also found that RGBS patients were younger, with milder disease and had Miller-Fisher variant of GBS at the initial episode. Patients with above characteristics on initial presentation of GBS are more prone for recurrences [3]. They also identified that there are similar presentations but more severe clinical deficit and residual effects with each recurrence [3, 6]. Yet, there is limited literature addressing why only a certain subset of patients with GBS get recurrences of the disease. The indexed case, although young at presentation, patient had a more alarming disease initially with poor neck muscle power and limb power and did not have Miller-Fisher variant. This shows a deviation from the classically identified features favoring a recurrence of GBS. The time gap between the episodes was 12 years. During the episode of recurrence, she had rapid development of more severe disabling illness involving cranial nerves and respiratory compromise needing mechanical ventilation. Both episodes had AIDP variant of GBS with similar initial presentations.

The RGBS patients with similar presentations during the subsequent episodes had different antecedent infections and this may point towards immunogenic and host factors as major determinants of the disease [3, 5, 6]. Yet, exact mechanism by which similar clinical manifestations occur during recurrence is not established. Our patient also had an upper respiratory tract infection preceding the recurrence of GBS but did not have an event during the initial episode.

It is important to distinguish RGBS from two clinical entities; (1) GBS with treatment related fluctuations (GBS-TRF), (2) Chronic inflammatory demyelinating polyneuropathy (CIDP). GBS-TRF which occurs in 6–16 % of patients with GBS is defined as significant deterioration within 2 months after disease onset following post treatment improvement or stabilization [3]. Repeating immunoglobulin or plasmaparesis in such patients will improve the outcome [10]. Since our patient had a long asymptomatic period, GBS-TRF is less likely but CIDP comes as a differential diagnosis. CIDP is suspected when progression of weakness lasts more than 8 weeks followed by a chronic course but it can be of steadily progressive, relapsing remitting or monophasic. The treatment differs as CIDP can be treated with either immunoglobulin or immunosuppressive therapy with a subsequent maintenance immunosuppressive drug treatment whereas GBS and GBS-TRF do not show a response to immunosuppressant therapy but has good response to immunoglobulin or plasmaparesis. GBS is a more likely diagnosis in our patient as there was a rapid onset of symptoms, subsequent complete or near complete recovery, high incidence of an antecedent illness, normal CSF protein levels at the onset of a recurrence.

Diagnosis of GBS is mainly clinical and supported by evidence from electrophysiological studies and CSF analysis. Characteristically CSF has high protein levels with normal cell counts and sugar levels. Nerve roots that exit from the spinal cord traverse through CSF and when nerve roots are inflamed in GBS, proteins leak in to the CSF. Since the inflammation is confined to the nerve roots, significant numbers of inflammatory cells are not seen. Although normal CSF findings are seen during the first week of disease, albuminocytologic dissociation is seen in 82–90 % of the patients with GBS by the end of second week of the illness [7]. Grand’maison et al. had also observed normal CSF proteins at the onset of recurrent GBS episodes but also noted that it was elevated when measured after 1 week and when the patient is symptomatic [4]. 10 % of patients with typical GBS may have normal CSF findings though out the course of illness [7, 8, 11] but the pathophysiological mechanism leading to normal values is not well understood. Medical literature on above area is not extensive. The index case also had normal levels of CSF total proteins (performed after 2 weeks of onset of illness) during both episodes of GBS, which is uncommon and not described in previously reported cases of RGBS.

Study done by Gonzalez-Quevedo et al. revealed raised CSF total proteins correlated with the degree of inflammation at the nerve roots and higher level of CSF proteins were related to clinical severity [12]. Corston et al. studied amino acids in CSF of GBS patients quantitatively as well as qualitatively. Of the 12 patients studied, 4 had normal CSF protein levels (<40 g/dL) and in 3 of them repeat CSF analysis confirmed normal CSF protein levels. Analysis revealed that 12 amino acids (Eg - ornithine, lysine, arginine, glycine, alanine plus citrulline, leucine, tyrosine and phenylalanine) were raised in patients with high CSF proteins while 6 of the amino acids (alanine plus citrulline, 2 amino-butyric acid, leucin, ornithine and lycine) were raised above normal reference range in patients who had normal CSF total proteins [13]. Three amino acids (phosphoethanolamine, serine and glutamic acid) in the CSF of GBS patients showed reduced concentrations. This study highlights the fact that even when the total protein content in CSF is normal, patients with GBS have alteration of specific amino acids in CSF. Our patient had a severe disease with respiratory compromise during the recurrence but in contrast to the study by Gonzalez-Quevedo et al. she had normal CSF protein levels. Due to the unavailability of the laboratory facilities, we could not perform the CSF amino acid analysis in our patient.

Index patient had a recurrence of GBS after a long asymptomatic period of 12 years as supported by acute onset, rapid progression, disability peaked within 2 weeks followed by complete subsequent recovery following treatment, presence of antecedent infection and supportive electrophysiological evidence. She also had more severe disease during the recurrence. CSF findings were not characteristic and were normal during both episodes occurring 12 years apart, which is a rare finding and not described earlier in patients with RGBS. Mechanism behind normal level of CSF proteins during recurrent episodes remains unclear.

Recurrence of GBS is rare but can occur after many years of asymptomatic period and is associated with more severe clinical manifestations. In such a presentation, it is important to distinguish GBS from CIDP as the treatment modalities are different. Normal CSF total protein levels tested after 1 week after the onset of disease can occur in initial and recurrent episodes of GBS, where the mechanism is not fully understood. Research on recurrent GBS is needed to evaluate the higher probability of certain subgroup of patients with GBS for recurrences, to identify the occurrence and pathophysiology of normal CSF profile and the possibility of a different subtype in recurrent GBS patients.

## Abbreviations

AIDP: Acute inflammatory demyelinating polyneuropathy
CIDP: Chronic inflammatory demyelinating polyneuropathy
CSF: Cerebrospinal fluid
GBS: Guillain-Barré syndrome
GBS-TRF: Guillain-Barré syndrome with treatment related fluctuations
RGBS: Recurrent Guillain-Barré syndrome
